# Statistical Assessment of the Effectiveness of Multiple COVID-19 Vaccinations on Daily Confirmed Cases in Seoul City

**DOI:** 10.7759/cureus.61457

**Published:** 2024-05-31

**Authors:** Jiwoo Kim, Hyosoon Jung

**Affiliations:** 1 Department of Medicine, University of Hawaii John A. Burns School of Medicine, Honolulu, USA; 2 Department of Preventive Medicine, Defense Health Agency, Area IV, Daegu, KOR

**Keywords:** vaccinated, confirmed cases, multiple dose, p-value, significance, regression, effectiveness of vaccination, covid-19 vaccination

## Abstract

This study investigates the effectiveness of multiple COVID-19 vaccinations on daily confirmed cases in Seoul City. Utilizing comprehensive data on vaccinated individuals and confirmed cases sourced from the official website of the Korean Ministry of the Interior and Safety, we conducted detailed statistical analyses to assess the impact of each vaccination dose. The study covers data from April 21, 2021, to September 29, 2022. Statistical multiple linear regression was employed to analyze the relationship between daily confirmed cases (positive outcomes from PCR tests) and multiple vaccine doses, using p-values as the criteria for determining the effectiveness of each dose. The analysis included data from four vaccination doses.

The analysis reveals that the first, second, and third doses of the COVID-19 vaccines have a statistically significant positive effect associated with the daily confirmed cases. However, the study finds that the fourth dose does not show a statistically significant impact on the reduction of daily confirmed cases. This suggests that while the initial three doses are crucial for establishing and maintaining high levels of immunity, the incremental benefit of subsequent doses may diminish.

## Introduction

The COVID-19 pandemic, which originated in Wuhan, China, in December 2019, rapidly escalated into a global crisis. By March 2020, the World Health Organization (WHO) had declared it a pandemic [1,2]. The virus's unprecedented scale and rapid spread posed significant challenges to healthcare systems worldwide. This crisis spurred global efforts to develop vaccines against COVID-19, marking a significant milestone in combating the virus. In response to the devastating impact of the virus, researchers and pharmaceutical companies swiftly developed and commercialized several significant vaccines against COVID-19 [3,4]. Among the first to receive emergency use authorization was Pfizer-BioNTech's BNT162b2, which demonstrated high efficacy rates in clinical trials [5,6]. Moderna's mRNA-1273 also showed robust efficacy in preventing COVID-19 infection [5]. Despite facing regulatory challenges and efficacy concerns, AstraZeneca's AZD1222, developed in collaboration with the University of Oxford, emerged as a promising candidate [5,6]. Similarly, Novavax's NVX-CoV2373 exhibited strong efficacy rates [5].

By December 2020, vaccination campaigns leveraging these vaccines were underway worldwide, offering hope for an eventual end to the pandemic. These campaigns aimed not only to immunize populations but also to foster herd immunity, protect vulnerable demographics, and alleviate the burden on healthcare infrastructures [7]. As vaccination efforts intensified, the WHO emphasized the importance of adhering to the recommended schedules for multiple vaccinations for each vaccine, crucial for optimal protection against COVID-19 and its variants [8]. In the meantime, questions have arisen regarding the necessity of multiple vaccinations [9,10].

However, significant challenges remain, including people's hesitancy to receive vaccinations and the need for strategic optimization in vaccination efforts [9]. This study addresses these issues by examining the effectiveness of multiple COVID-19 vaccinations on daily confirmed cases in Seoul City. We chose Seoul City as the focus of this study because South Korea exhibits one of the highest vaccination rates globally, for both initial and booster doses [11]. Therefore, Seoul City provides an optimal population for investigating the impact of vaccination on COVID-19 case dynamics. By leveraging comprehensive datasets on vaccination status and daily confirmed cases, this research aims to analyze trends and associations at a macro level. Such an ecological study design provides valuable insights into the public health impact of vaccinations, informing strategies to enhance vaccine uptake and optimize the allocation of resources.

Our objective throughout these multiple linear regression analyses was to discern the extent to which variations in vaccine dosing correlate with fluctuations in daily confirmed cases. By systematically evaluating the statistical significance of each dose, we aimed to provide valuable insights into the effectiveness of multiple COVID-19 vaccinations in Seoul City. This analysis was instrumental in identifying the doses that had the most substantial impact on daily confirmed cases and in understanding the overall effectiveness of the vaccination campaign. The insights derived from this statistical evaluation can inform public health strategies and vaccination policies.

The primary aim of this study is the statistical analysis of these datasets to assess the effectiveness of each vaccine dose in influencing daily confirmed COVID-19 cases.

## Materials and methods

Data collection and integration

To comprehensively evaluate the impact of multiple COVID-19 vaccination doses on daily confirmed cases in Seoul City, we meticulously conducted statistical analyses using robust methodologies [12]. Our approach leveraged datasets provided by the Korean Ministry of the Interior and Safety, which included detailed information on vaccination rates and daily confirmed COVID-19 cases.

We procured two pivotal datasets essential for our analysis: "Confirmed Cases of COVID-19 in Seoul City" and "COVID-19 Vaccination Status in Seoul City." These datasets were sourced from the official website of the Korean Ministry of the Interior and Safety [13]. Confirmed cases are individuals classified as having COVID-19 if they have tested positive for the SARS-CoV-2 virus using a PCR test, which detects the genetic material (RNA) of the virus. Through careful data integration, we merged these datasets into a unified set conducive to statistical analysis using Minitab software. This integration process involved aligning the data points chronologically and ensuring consistency in reporting formats. Table 1 exemplifies sample rows extracted from this integrated dataset, offering insights into the structured data representation.

**Table 1:** Cumulative data on individuals vaccinated with multiple doses and daily confirmed COVID-19 cases from April 21, 2022, to March 15, 2023, covering 10 days of data, as an example.

Date	First dose	Second dose	Third dose	Fourth dose	Daily confirmed cases
10-Mar-22	8309642	8222838	5764351	-	138
11-Mar-22	8311183	8225143	5779624	-	140
12-Mar-22	8311680	8225806	5786263	-	140
13-Mar-22	8311681	8225811	5786401	-	112
14-Mar-22	8312602	8226915	5795463	-	112
15-Mar-22	8313033	8227560	5801689	17539	79
16-Mar-22	8313346	8227846	5807312	19227	120
17-Mar-22	8313864	8228431	5816025	20739	124
18-Mar-22	8314802	8229479	5830011	22944	146
19-Mar-22	8315182	8229848	5835718	23396	120

Study design

This study employs an ecological study design. An ecological study focuses on vaccine-eligible populations, which numbered 9,505,868 as of September 23, 2021, in Seoul City rather than individuals. This approach is suitable for analyzing the overall impact of vaccination doses on the daily confirmed cases of COVID-19 at the city level. By leveraging aggregated data on vaccination rates and confirmed cases, this approach allows for the examination of trends and associations at a macro level, providing valuable insights into the public health impact of vaccinations.

Study period

Our analysis spanned a crucial timeline capturing the evolution of COVID-19 vaccination efforts in Seoul City. Commencing with the administration of the first vaccine dose on February 26, 2021, data collection ensued for both the first and second dose vaccinations alongside daily confirmed cases from April 21, 2021. Subsequently, data on third dose vaccinations were aggregated from October 13, 2021, followed by the inclusion of fourth dose data from March 15, 2022. The study encompassed all individuals within Seoul City who received COVID-19 vaccinations during these specified periods.

In statistical multiple linear regression, the initial several months of each dose were focused on to utilize the rapidly changing slopes of vaccinated individuals. The first and second doses were calculated from April 21, 2021, to October 12, 2021; the third dose was from October 13, 2021, to December 30, 2021; and the fourth dose was from March 14, 2022, to September 29, 2022. By focusing on these initial periods, we aimed to capture the most dynamic phase of vaccination uptake and its immediate effects on confirmed case numbers. This approach allowed us to better understand the temporal relationship between vaccination rates and the incidence of COVID-19.

Ethical considerations

This study was conducted using publicly available, anonymized data from the Korean Ministry of the Interior and Safety. As the data did not contain any personally identifiable information, and the research did not involve direct interaction with human subjects, formal ethical approval was not required. However, all procedures were conducted in accordance with the guidelines and regulations set forth by the relevant institutional and national research committees.

Statistical analysis

To assess the efficacy of each vaccine dose in mitigating daily confirmed COVID-19 cases, we adopted a robust statistical technique: multiple linear regression analysis. Despite all variables being discrete, multiple linear regression can be utilized for this study because the number of vaccinations and daily confirmed cases have an approximately linear relationship. This analytical approach facilitated the modeling of the relationship between vaccination doses and daily confirmed cases, with daily confirmed cases serving as the dependent variable and vaccination doses as independent variables. To focus on the rapidly changing slopes of vaccinated individuals at the initial stage of each vaccination, multiple linear regression analyses were conducted several times.

## Results

**Figure 1:**
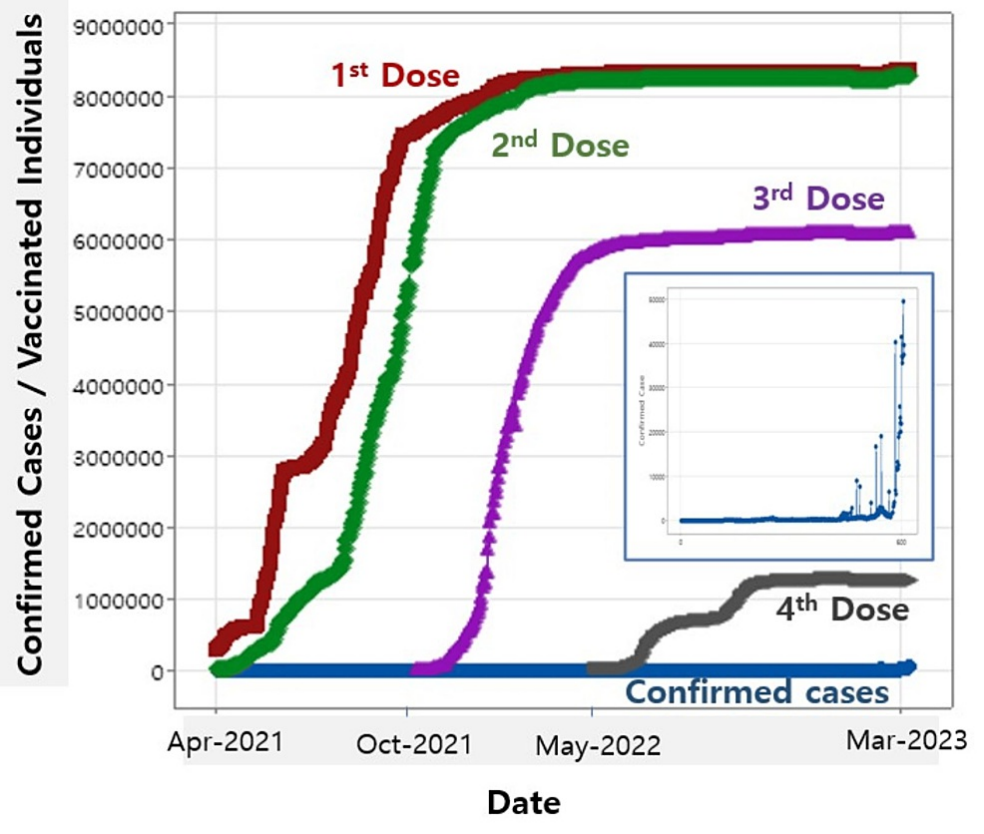
Vaccinated individuals and daily confirmed cases across multiple vaccination doses. The inset highlights the daily confirmed cases.

The data on vaccinated individuals across four doses and daily confirmed cases are presented in Figure 1, with the daily confirmed cases distinctively illustrated in the inset. The data in the figure spans from April 21, 2021, to March 15, 2023.

**Figure 2:**
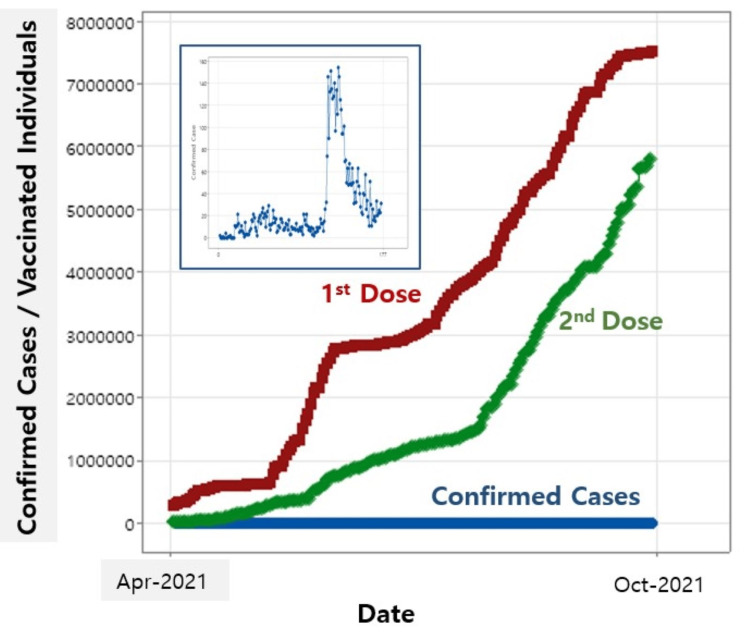
Vaccinated individuals for the first and second doses with daily confirmed cases. The inset highlights the daily confirmed cases.

In order to focus on the initial increase in vaccinated individuals receiving the first and second doses, the date range from April 21, 2021, to October 21, 2021, was chosen, as shown in Figure 2. Despite differing start dates for the first and second doses, data on vaccinated individuals for these doses were compiled starting from the same date alongside the daily confirmed cases. The inset clearly highlights the daily confirmed cases. To determine the effectiveness of vaccinations on daily confirmed cases, a statistical regression analysis was conducted. The analysis used the first and second vaccine doses as independent variables and daily confirmed cases as the dependent variable, covering the same date range shown in Figure 2.

The results of this analysis are displayed in Table 2.

**Table 2:** Regression results for the first and second doses with daily confirmed cases. Coef: coefficient; SE coef: standard error of the coefficient; R-sq: R-squared (the "R" in "R-squared" stands for the "correlation coefficient"); R-sq (adj): adjusted R-squared; Adj SS: adjusted sum of squares; Adj MS: adjusted mean square

Coefficient				
Term	Coef	SE coef	T-value	P-value
Constant	-8.53	5.98	-1.43	0.156
First dose	0.000019	0.000004	4.46	0.000
Second dose	-0.000017	0.000006	-2.82	0.005
Model summary				
R-sq	R-sq (adj)	-	-	-
24.58%	23.70%	-	-	-
Analysis of variance				
Source	Adj SS	Adj MS	F-value	P-value
Regression	57048	28524	27.87	0.000
First dose	20336	20336	19.87	0.000
Second dose	8121	8121	7.94	0.005

The regression analysis yielded the following equation: daily confirmed cases = 8.53 + 0.000019 first dose - 0.000017 second dose (Equation 1).

In addition to obtaining the regression equation, the p-values shown in Table 2 are 0.000 for the regression model, 0.000 for the first dose, and 0.005 for the second dose. The regression result clarifies that the first and second doses have positive impacts on the daily confirmed cases.

**Figure 3:**
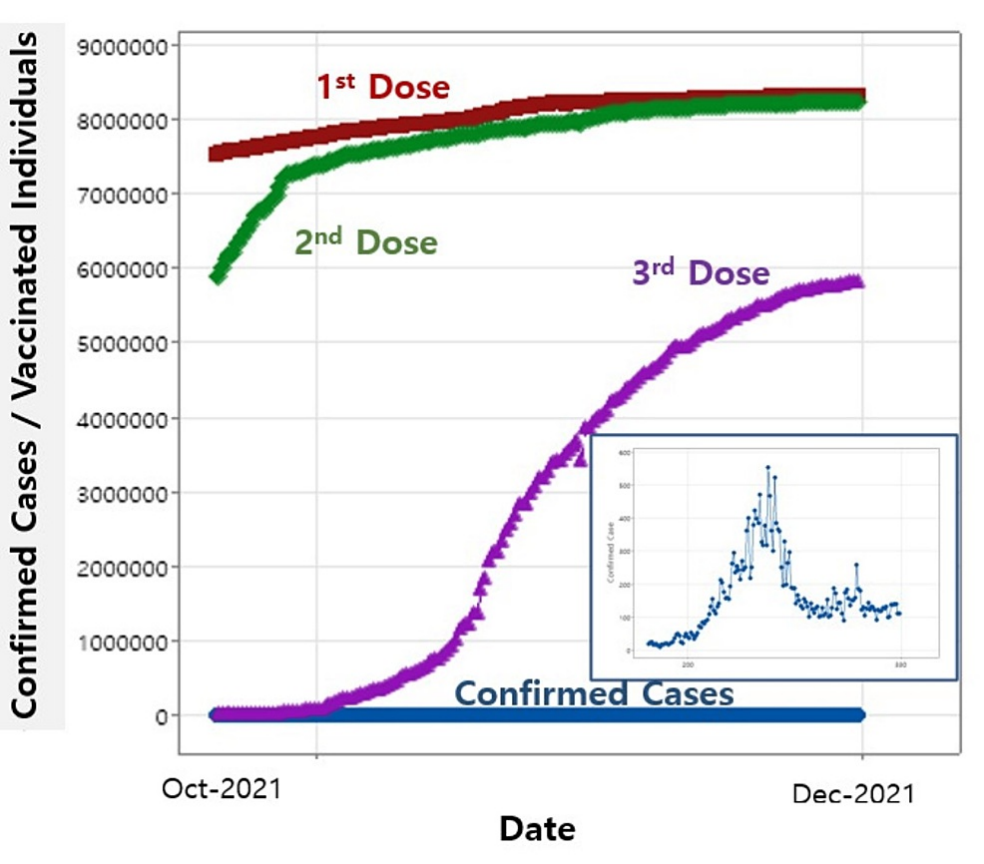
Vaccinated individuals for the first, second, and third doses with daily confirmed cases. The inset highlights the daily confirmed cases.

Similarly, a regression analysis incorporating an additional third dose is conducted. The data on vaccinated individuals for the first, second, and third doses, along with daily confirmed cases, are shown in Figure 3.

The inset emphasizes the daily confirmed cases around the initial rollout of the third dose, on which the regression focuses. The regression equation of the three doses with the daily confirmed cases is as follows: daily confirmed cases = -12,913 + 0.001964 first dose - 0.000306 second dose - 0.000134 third dose (Equation 2).

The regression results summarized in Table 3 reveal all four p-values to be 0.000 for both the regression model and each dose. This indicates a significant regression model and a strong impact of the first, second, and third doses on daily confirmed cases.

**Table 3:** Regression results of the first, second, and third doses with daily confirmed cases. Coef: coefficient; SE coef: standard error of the coefficient; R-sq: R-squared (the "R" in "R-squared" stands for the "correlation coefficient"); R-sq (adj): adjusted R-squared; Adj SS: adjusted sum of squares; Adj MS: adjusted mean square

Coefficient				
Term	Coef	SE coef	T-value	P-value
Constant	-12913	842	-15.33	0.000
First dose	0.001964	0.000138	14.19	0.000
Second dose	-0.000306	0.000038	-8.13	0.000
Third dose	-0.000134	0.000009	-15.35	0.000
Model summary				
R-sq	R-sq (adj)	-	-	-
67.49%	66.84%	-	-	-
Analysis of variance				
Source	Adj SS	Adj MS	F-value	P-value
Regression	1314471	438157	103.81	0.000
First dose	849688	849688	201.31	0.000
Second dose	278946	278946	66.09	0.000
Third dose	994698	994698	235.67	0.000

The R-square value from this regression suggests that the model explains approximately 67% of the variance in daily confirmed cases [14]. The R-square value in this regression is higher than that of the previous regression with the first and second doses in Table 2, and the third dose clearly contributes to the daily confirmed cases.

**Figure 4:**
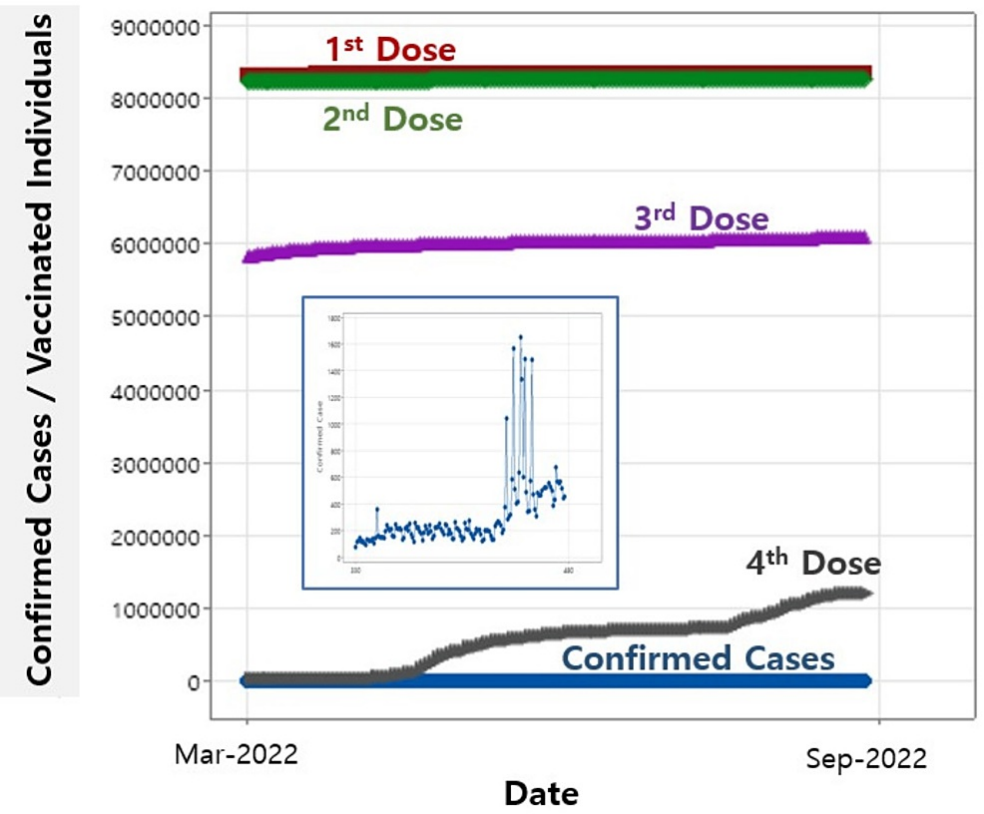
Vaccinated individuals for the first, second, third, and fourth doses with daily confirmed cases. The inset highlights the daily confirmed cases.

Throughout the two previous regression analyses, it is noteworthy that the first, second, and third doses show significance and have positive impacts on daily confirmed cases. The subsequent analysis extends to the fourth dose, as illustrated in Figure 4, where the data spans from March 15, 2022, to September 15, 2022, showing the vaccinated individuals of all four vaccination doses with daily confirmed cases. Detailed variation of the daily confirmed cases is shown in the inset.

The regression results, outlined in Table 4, demonstrate the regression equation: daily confirmed cases = 6,572 - 0.00251 first dose + 0.00108 second dose + 0.000939 third dose - 0.000079 fourth dose (Equation 3).

**Table 4:** Regression results of the first, second, third, and fourth doses with daily confirmed cases. Coef: coefficient; SE coef: standard error of the coefficient; R-sq: R-squared (the "R" in "R-squared" stands for the "correlation coefficient"); R-sq (adj): adjusted R-squared; Adj SS: adjusted sum of squares; Adj MS: adjusted mean square

Coefficient				
Term	Coef	SE coef	T-value	P-value
Constant	6572	44602	0.15	0.883
First dose	-0.00251	0.0059	-0.43	0.671
Second dose	0.00108	0.00286	0.38	0.706
Third dose	0.000939	0.000701	1.34	0.184
Fourth dose	-0.000079	0.00004	-1.98	0.051
Model summary				
R-sq	R-sq (adj)	-	-	-
24.39%	21.39%	-	-	-
Analysis of variance				
Source	Adj SS	Adj MS	F-value	P-value
Regression	68985	17246.3	8.14	0.000
First dose	383	383.2	0.18	0.671
Second dose	303	303.3	0.14	0.706
Third dose	3795	3795.5	1.79	0.184
Fourth dose	8278	8278.4	3.91	0.051

The p-values explicitly indicate that while the regression analysis remains significant, the four doses do not contribute significantly to the daily confirmed cases, in which discrepancy is seen among the significances of the regression model and each dose.

## Discussion

Both the numbers of individuals receiving the first and second vaccine doses exhibit similar trends, particularly at saturation levels of 88.6% and 87.8%, respectively, as shown in Figure 1. The vaccination rates indicate that vaccine hesitancy among the people in South Korea appears to be quite low compared to other countries [15]. The high rate of adherence to the second vaccination reflects commendable behavior among vaccinated individuals, given that the positive effects of the second vaccination have been demonstrated [9,16,17]. However, the vaccination rates for the third and fourth doses decline significantly, with saturation points at 64.6% and 13.4%, respectively. This suggests a general reluctance among vaccine-eligible individuals to pursue multiple vaccinations, particularly concerning the booster shots of the third and fourth doses. It is plausible that once a significant portion of the population receives two doses, the perceived urgency for additional doses decreases, leading to lower uptake rates for the third and fourth doses. Additionally, hesitations and concerns about receiving multiple COVID-19 vaccinations have arisen [9,15]. Some individuals have expressed apprehensions about the safety of receiving multiple doses of COVID-19 vaccines, particularly regarding potential side effects or long-term health consequences [18]. Furthermore, inquiries have emerged concerning the efficacy of booster doses in providing additional protection against COVID-19, especially in light of emerging variants [9,18,19].

Previous research by Chenchula et al. has demonstrated the necessity and positive effects of a third dose [19]. Positive effects of a fourth vaccine dose against severe illness caused by the Omicron variant and in reducing the short-term risk of COVID-19-related outcomes have also been observed [20,21].

It should be noted that our statistical assessment indicates that the third dose actually contributes to the reduction of daily confirmed cases. Therefore, it appears necessary to assess the effectiveness of a fourth dose, given that the positive effects of the first, second, and third doses have been confirmed.

As for the regression results with the fourth dose in Table 4 with the regression (Equation 3), the p-values for all four doses are greater than 0.05, suggesting that the fourth dose is not effective in reducing daily confirmed cases. To elucidate the effect of the fourth dose, an additional regression analysis was conducted, maintaining all previous regression conditions, including the date range covered, except for the removal of the fourth dose. The resultant regression equation is as follows: daily confirmed cases = - 11,510 + 0.001750 first dose - 0.000272 second dose - 0.000110 third dose (Equation 4).

All p-values from the regression model for the first, second, and third doses are 0.000, confirming that the fourth dose is not statistically significant and not effective in reducing daily confirmed cases. These results are similar to the previous regression (Equation 2), which also used the same independent variables of the first, second, and third doses. The only difference between the two regressions with Equations 2 and 4 is the date range involved in the calculation; Equation 2 was calculated over the same date range as shown in Figure 3, while Equation 4 used the range seen in Figure 4.

## Conclusions

Statistical regression analysis yielded valuable insights into the effectiveness of multiple COVID-19 vaccinations concerning daily confirmed cases in Seoul City. We found that the first, second, and third doses are statistically significant and positively effective on daily confirmed cases of COVID-19, as indicated by the regression analyses. However, our analysis revealed that the fourth dose did not exhibit any effectiveness on daily confirmed cases. The determination of the effectiveness of multiple vaccinations relied on the p-values derived from the regression analyses.
